# Short-term progression of optic disc and macular changes in optic nerve head drusen

**DOI:** 10.1038/s41433-022-02155-7

**Published:** 2022-07-16

**Authors:** Anastasia V. Pilat, Frank A. Proudlock, Periyasamy Kumar, Irene Gottlob

**Affiliations:** 1 Ophthalmology Group, University of Leicester, Leicester, UK; 2 East Sussex NHS Healthcare Trust, Sussex, UK; 3 Leicester Royal Infirmary, Leicester, UK; 4 Cooper Medical School of Rowan University and Cooper University Healthcare, Camden, NJ USA

**Keywords:** Eye abnormalities, Sensory systems

## Abstract

**Purpose:**

To quantify in patients with optic nerve head drusen (ONHD)changes after 1-year observation in: (i) optic disc and (ii) macular optical coherence tomography (OCT) parameters and (iii) the effect of age at enrolment in the study.

**Design:**

Prospective, cross-sectional observational study using Spectral Domain-OCT (Copernicus; OPTOPOL Technology S.A., Zawiercie, Poland) imaging was carried out in 35 patients with ONHD (age–42.8 ± 19.9 years; males = 15; females = 20) at baseline and after 12 months follow-up.

**Results:**

Patients with ONHD had significant thinning of the surface nerve fibre layer in the central (*p* = 0.03), superior (*p* = 0.05) and inferior (*p* = 0.04) areas; mean ppRNFL thinning (*p* = 0.0 4) and ppRNFL thinning in the nasal segment (*p* = 0.028). Retinal thinning in the central (*p* = 0.001), inner (*p* = 0.01) and outer (*p* = 0.002) temporal, outer superior (*p* = 0.03) and inferior (*p* = 0.02) areas; borderline ganglion cell layer thinning (*p* = 0.051) and outer nuclear layer (*p* = 0.03) thinning in the central retina and outer segment layer thinning nasally (*p* = 0.01) between the first and the second visit in macula. Correlation of the difference in optic disc and macular parameters with the age at enrolment did not reveal any significance.

**Conclusions:**

Statistically detectable thinning of the optic nerve and macula structures occurred already after 12 months. The proximity of optic nerve changes to the vascular arcades can possibly be explained by involvement of retinal vessels in the pathophysiology of ONHD.

## Introduction

Optic nerve head drusen (ONHD) is a relatively common condition with an incidence from 0.34 to 2.4% in the population [1–5]. The incidence is likely to be underestimated as patients with ONHD are often asymptomatic. About 60% of patients have deeply located (buried) ONHD making their detection by fundoscopy and differentiation from optic disk oedema difficult [6]. Based on histochemical studies, MO postulated in 1981 [7] that the origin of ONHD is related to axoplasmic derivatives of disintegrating nerve fibres (degenerated axons). The chemical composition of the optic nerve drusen consists of mucopolysaccharides, ribonucleic and deoxyribonucleic acids, amino acids, calcium, and a small amount of iron [8]. In 2008, tissues obtained after vitrectomy from patients with ONHD analysed with energy-dispersive spectroscopy suggested that Ca^3^(PO4)^2^ is the main salt present in ONHD [9]. Due to the presence of calcium, autofluorescence and ocular ultrasound were the most useful techniques in clinical practice before the availability of optical coherence tomography (OCT). Autofluorescence can detect some of the deeply located drusen with higher sensitivity then ophthalmoscopy [6].

Recently, OCT has been widely used in clinical practice for the detection of ONHD. ONHD have been described in some studies as subretinal hyperreflective peripapillary ovoid areas located in the outer plexiform and outer and inner nuclear layers above the edges of the RPE [10].

A number of recent OCT studies by Malmquist et al. including publications by Optic Disc Drusen Studies Consortium, describe ONHD as a hypo-reflective structures often seen with a hyper-reflexive margin unlike previously reported. The peripapillary peripapillary hyperreflective ovoid mass-like structures (PHOMS) are often associated with drusen [11–15]. It is suggested that PHOMS are non-specific signs of the axonal distension and crowding that can be present in both congenital and acquired neuropathies [16, 17].

The value of the thickness of the peripapillary retinal nerve fibre layer for the differential diagnosis of ONHD and ODE is controversial. Optic atrophy with RNFL thinning and RNFL elevation as well as RNFL thickening has been described in both groups of patients by different authors [18–20]. Nevertheless, most authors agree that thickening of RNFL can help to diagnose ODE and differentiate from ONHD when the thickness is severely increased as compared to the healthy population. Moreover, mild retinal changes including intraretinal fluid accumulation, ‘folding’ of the retinal pigment epithelium in oedema [21] and thinning of the inner plexiform, nerve fibre layer were reported in oedema patients.

Several research groups reported in cross-sectional studies that ONHD increase in size with age on funduscopic examination [8, 22, 23]. In young patients drusen are often not clearly visible appearing as uniformly elevated optic discs because drusen are buried deep inside the papilla [24]. A few reports have recorded longitudinal fundus images and describe increase in size of drusen with age during a period of 5–15 years usually without any changes in visual fields or visual acuity [25, 26]. A recent publication by Malmqvist et al. [14] based on the fundus photography of 8 patients initially examined by Lorenzen in 1958–1959 with median follow-up 56 years found minimal progression of the number and volume of ONHD, more obvious in younger patients [27].

OCT is a standard non-invasive clinically available test with high potential to objectively assess optic nerve head and macular structure and has not been used for objective longitudinal assessment of progression of the size of drusen to date. In this study we aim to objectively investigate whether drusen cause progression of the optic nerve and retina structural changes over a short time. We measured differences in: (i) optic disc and (ii) macular OCT parameters in patients with ONHD after 12 months observation using spectral-domain optical coherence tomography (SD-OCT). We also explored the effect of age on these changes.

## Participants and methods

The population cohort for this prospective observational study included 35 patients with bilateral ONHD (aged between 8 and 74 years old; mean = 42.8, SD = 19.8); males = 15; females = 20, Supplementary Table 1).

Initial clinical examination for all participants included best-corrected visual acuity, intraocular pressure measurements; visual field testing (24–2 pattern, Humphrey Field Analyser, Carl Zeiss Meditec, normal in all patients, performed at the 1st exam), Ishihara colour vision test, eye ultrasound and fundus photography with filter for autofluorescence. Fluorescein angiography, magnetic resonance imaging and lumbar puncture were performed, if necessary, to exclude patients with co-existing optic disc oedema secondary to high intracranial pressure.

The diagnosis of ONHD was established based on the criteria previously used in other publications investigating ONHD [14, 18]. All patients had visible ONHD on fundus examination, autofluorescence, calcification on ultrasound of the ONH, normal opening cerebrospinal fluid pressure if lumbar puncture was performed and absence of visibly apparent progressive protrusion of the optic nerve on follow-up examination by OCT. All participants had full colour vision on Ishihara testing and no changes on Humphrey Visual Field testing and normal intraocular pressure (mean = 16.5, SD = 2.0 mm). Patients with visual field defects have been excluded from the study to eliminate the possibility of the co-existing neuropathy. They had no other eye conditions or systemic disease, no previous intraocular or refractive surgery.

Refractive error was within ±3.0 D to reduce the risk of errors of lateral distance measures caused by changing axial lengths.

All participants had OCT measurements on initial visit (1st visit) and in 12 months (±1 week) follow-up visit (2nd visit).

The study was approved by the local ethics committee and adhered to the tenets of the Declaration of Helsinki. Each participant or parents/guardians of participants of this study gave informed consent.

### Optical coherence tomography

Spectral-domain OCT (Copernicus; OPTOPOL Technology S.A., Zawiercie, Poland) with a theoretical axial resolution of 3 μm and a transverse resolution of 12 μm (75 B-scans, 7 × 7 × 2 mm volume, each B-scan consisted of 743 A-scans) was used to measure optic nerve and macular parameters of patients with bilateral ONHD. The best quality scan (no motion/blinking artefacts, signal strength ≥6) from one eye was selected at the initial examination (visit 1) and compared to the scan from the same eye after 12 months follow-up (visit 2). We were not able to use the automated recognition of the RNFL due to difficulty of automated segmentation in drusen. Analysis and adjustment of the automated recognition when necessary were performed by the same investigator (AP) who was masked to the name of the patient and the number of the visit to minimise possible inaccuracies.

#### Optic disc parameters

Due to the often deep location of the drusen within nerve head and limitation of the OCT scan depth range we were not able to assess changes of the drusen in all patients. Therefore, disc protrusion and thickness of the hyperreflective fibre layer was done to elevate the difference of the head protrusion and thickness of the nerve fibres.

##### Optic disc protrusion

The standard automated optic disc OCT protocol is mainly focused on cup diameter/depth when patients have obvious detectable cup, whereas patients with drusen often do not have cup; therefore, conventional optic disc protocol does not allow to provide disc protrusion measurements of the patients with drusen.

**Fig. 1:**
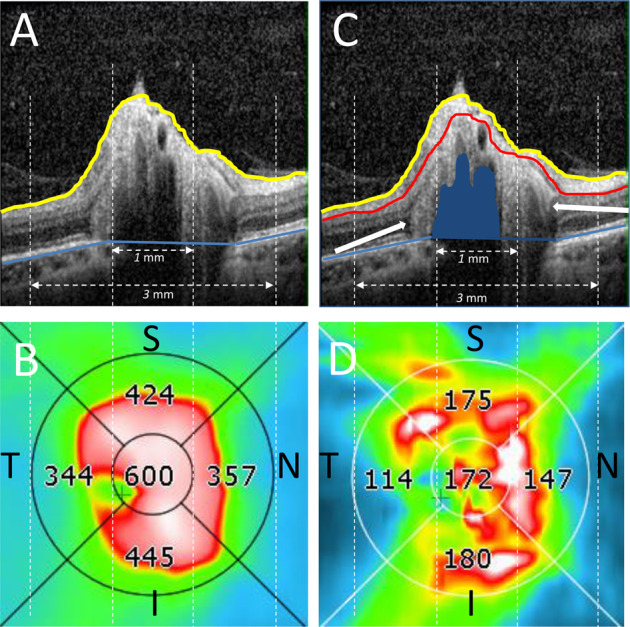
Analysis of optic disc protrusion achieved by applying the macula protocol to the optic disc. **A** B-scan through the centre of the right optic disc of a participant showing the delineation of the measurement of optic disc protrusion consisting of the area between the internal limiting membrane (yellow line) and the retinal pigment epithelium (blue line). **B** Thickness measurements of the optic nerve head protrusion in in the central circular 1 mm and 3 mm areas. **C** Same B-scan as (**A**) showing surface nerve fibre layer (hyperreflective layer between yellow and red lines). The white arrows show the peripapillary hyperreflective ovoid mass-like bodies. The hypo-reflective area on (**A**) corresponding to the shape outlined in blue on (**B**) is showing the position of drusen. **D** Thickness of the hyperreflective layer in the central circular 1 and 3 mm areas. S superior, I inferior, N nasal, T temporal segments. Colours of the images (**B**, **D**): blue = 200–280 µm, green = 280–400 µm, yellow = 400–500 µm, red = over 500 µm.

To avoid potentially spurious values dependant on the disc area measurements, we modified a standard macular protocol (Copernicus OCT software) for use on the optic nerve head region which generates thickness measurements over fixed areas of the papillary region: (i) a central 1 mm diameter region, and (ii) a 1–3 mm diameter annulus which is divided into four quadrants, superior, inferior, nasal and temporal (see Fig. 1). To ensure consistency between repeated measurements the centre point of the measurement was placed at the centre of the disc defined as midway between the horizontal and vertical disc diameter measurements.

The segmentation protocol assesses mean thickness from the internal limiting membrane to the Bruch’s membrane for these defined areas providing a measure of the optic disc protrusion. The missing portion of Bruch’s membrane at the optic nerve disc is fitted with a spline function based on surrounding visible Bruch’s membrane measurements. Although not an anatomically true nerve fibre layer measurement the hyperreflective layer on the optic nerve head protrusion has the similar reflectance profile on the OCT image as the retinal nerve fibre layer and hence could be segmented consistently using a retinal nerve fibre layer algorithm for the same defined areas. We termed this measurement the surface nerve fibre layer (hyperreflective space). The results derived from the segmentation of other retinal layers using this protocol was ignored. The segmentation lines derived from automatic segmentation for the internal limiting membrane, Bruch’s membrane and lower surface of the hyperreflective space were verified and corrected manually where necessary over the region of interest.

Because this is a newly developed protocol we carried out a repeatability analysis in 13 participants on whom repeated scans have been carried out on the same visit (see Supplementary Table 2). The analysis demonstrates extremely high repeatability for all parameters with ICCs of >0.998 or better and upper and lower limits of agreement of <6 µm around the mean difference.

##### Peripapillary RNFL

Peripapillary RNFL (ppRNFL) thickness was measured within the temporal, superior, nasal and inferior quadrants of an annulus of 0.4 mm width with an internal diameter of 2.4 mm (default settings of automatic programme). Mean ppRNFL thickness (averaged between the quadrants) was also measured. The position of the internal limiting membrane and RNFL were delineated using automated algorithms in the software and manually corrected by one investigator to correct any segmentation errors.

#### Macular parameters

##### Retinal and RNFL thickness

Macular and RNFL thickness in the macular area were measured using the manufacturer’s software. The position of the internal limiting membrane, the outer limit of the RNFL and the RPE were delineated using automated algorithms in the software. Segmentation errors were manually corrected. The retinal and RNFL thickness were measured in 3 circular zones as defined by EDTRS protocol [27] consisting of a central annulus (1 mm), inner annulus (1 to 3 mm) and outer annulus (3 to 6 mm), the inner and outer annuli were also divided into four separate segments (superior, inferior, temporal and nasal).

##### Retinal structural analysis

For the detailed assessment of the changes in individual retinal layers a central horizontal flattened B-scan was used for each participant to measure the individual layer thicknesses using an ImageJ macro. ImageJ macro automatically generated ten points in five locations of the fovea (central, 1 mm and 3 mm temporarily and nasally from the centre) using the protocol defined by Mohammad et al. [28] and used for thickness measurements of the retinal nerve fibre (RNFL), ganglion cell layer (GCL), inner plexiform, inner nuclear, outer plexiform, outer nuclear (ONL), inner segment, outer segment (OS) and retinal pigment epithelium (RPE) layers.

##### Statistical methods

Statistical analysis was carried out using SPSS 24 software (SPSS, Inc., Chicago, IL). Original data of all parameters was normally distributed as tested by the Shapiro–Wilk test.

Paired *t* tests were used to calculate the differences in different OCT parameters after 12 months in the same patients (1st vs. 2nd visits) with age as fixed factor to assess its impact on progression. Bonferroni correction was used for multiple comparisons. Correlation analysis was performed using Pearson’s, coefficient of correlation. *P* ≤ 0.05 was considered statistically significant.

## Results

Patients with ONHD did not show any changes in visual acuity and visual field test results on follow-up visit.

### Optic nerve head

**Fig. 2:**
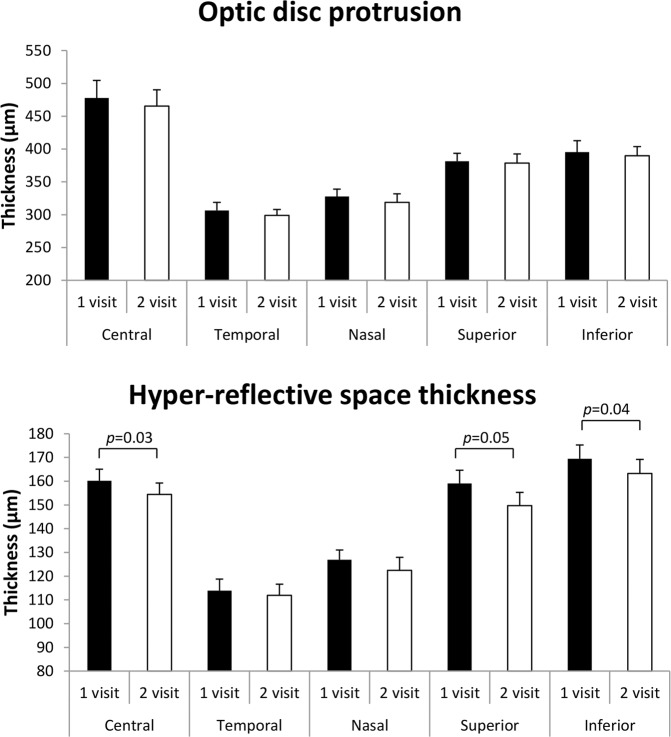
Thickness of optic nerve head protrusion and thickness of the surface nerve fibre layer (µm) in patients with optic nerve head drusen: initial and 12 months follow-up visit.

Patients did not demonstrate any significant difference in the thickness of the optic disc protrusion on the second visit. By contrast, the surface nerve fibre layer over the optic disc was statistically significantly thinner in the central (*t* = −2.15; *p* = 0.03), superior (*t* = −2.21; *p* = 0.05) and inferior (*t* = −2.39; *p* = 0.04) areas at the second visit (Fig. 2, Supplementary Figs. 1, 2).

**Fig. 3:**
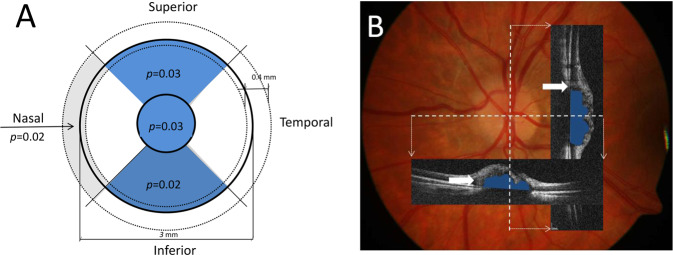
Changes in peripapillary retinal nerve fibre layer (ppRNFL) and surface nerve fibre layer thickness in patients with optic nerve head drusen (ONHD). **A** Schematic diagram showing the optic disc areas with significant thinning in the surface nerve fibre layer (dark blue sectors) and ppRNFL (light grey quarter-annulus between dotted circles) between the first and second visit; continues circles show the area of the optic disc where the protrusion and surface nerve fibre layer thickness was measured using the macula protocol; dotted circles = measurements for RNFL thickness protocol; (**B**) Fundus photography of the left eye of a patient with ONHD. Areas with significant thinning (**A**) match the location of large blood vessels in the fundus (**B**). Horizontal and vertical OCT tomograms (corresponding to location of white hatched lines) show the presence of ONHD (blue area) and peripapillary hyperreflective ovoid mass-like bodies (white arrows).

The changes in ppRNFL thickness also showed significant thinning in the mean ppRNFL (*p* = 0.004) and the nasal (*p* = 0.028, Fig. 3, Supplementary Fig. 2) segment. Thinning was also close to significance in the temporal segment (*p* = 0.053, Supplementary Fig. 2).

### Macular parameters

Between the first and second examination significant thinning of the retina in patients with drusen was found in the central (*p* = 0.001), inner (*p* = 0.01) and outer (*p* = 0.002) temporal, outer superior (*p* = 0.03) and inferior (*p* = 0.02) areas.

**Fig. 4:**
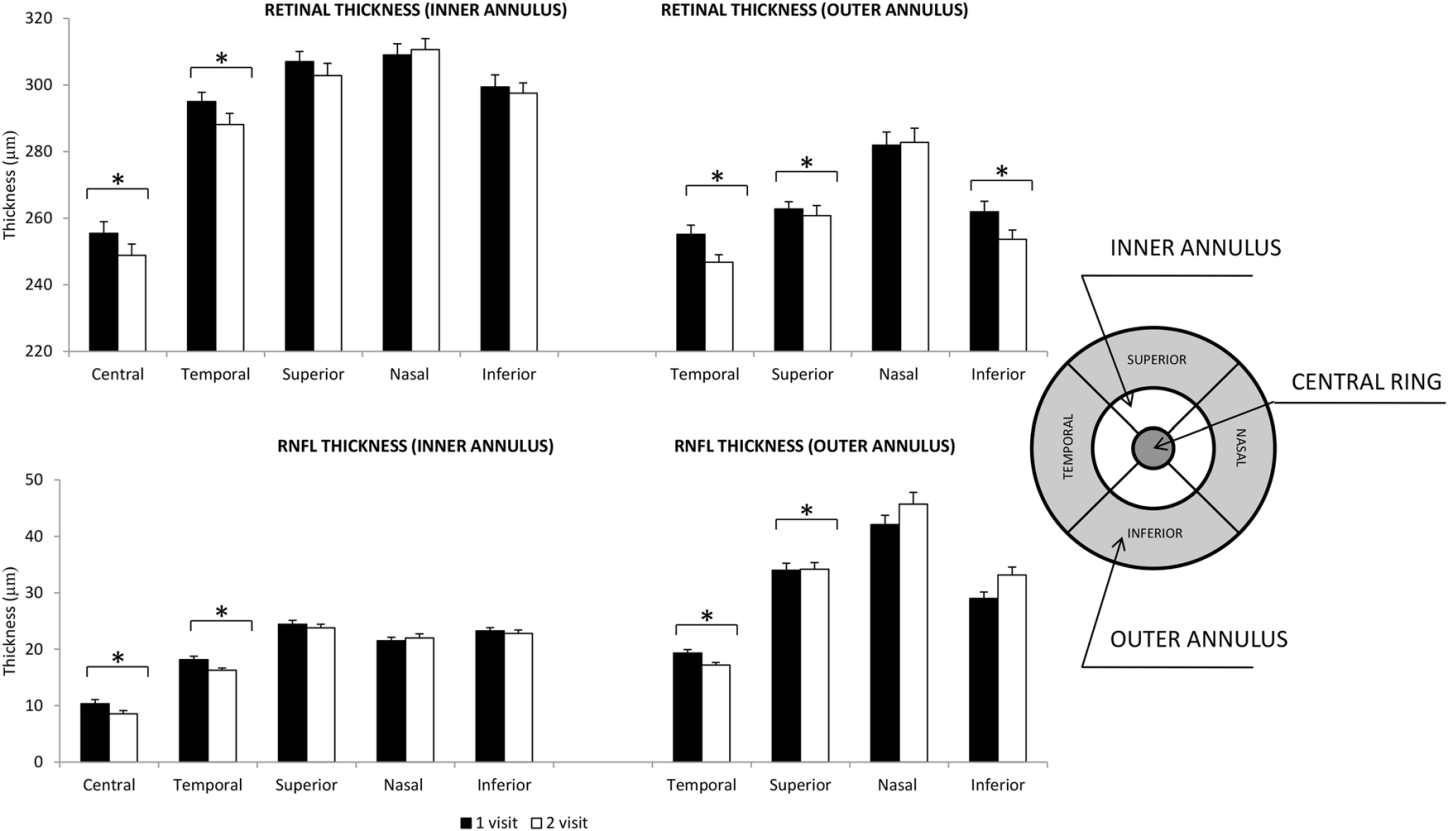
Changes in average retinal and retinal nerve fibre layer thickness (RNFL, µm) in the macular area in patients with optic nerve head drusen. Asterisk (*) indicates significant difference (*p* ≤ 0.05).

In the central and temporal areas there was thinning of the macular RNFL (*p* = 0.04, *p* = 0.02 and *p* = 0.04 for the central and temporal inner and outer annulus respectively, Fig. 4).

### Retinal structural analysis

Subanalysis of the individual retinal layer thickness at the macula indicated statistically significant thinning of RNFL (*p* = 0.01) and ONL (*p* = 0.03) in the central retina and OS layer nasally (*p* = 0.01) between the first and the second visit. Difference in GCL was close to significance (*p* = 0.051).

### Age effect on the optic disc and fovea

There was a significant negative correlation of the patients’ age with the absolute value of the mean ppRNFL thickness (*p* = 0.003), the superior hyperreflective space (*p* = 0.01) and the central retinal thickness (*p* = 0.02). In contrast, the correlation of the difference between the first and second visit with age at enrolment did not reveal significance for any of the parameters.

## Discussion

This is to our knowledge the first quantitative study investigating optic nerve and macular changes over time in patients with ONHD using OCT. We found significant reduction in the mean and nasal ppRNFL thickness with thinning of the surface nerve fibre layer *(*hyperreflective space) over the optic nerve head centrally, superiorly and inferiorly. In the macula area we also found retinal thinning caused by and RNFL, ONL and OS thinning. Statistically detected changes occurred in a short time over only 1 year.

### Optic nerve head measurements

Total optic disc prominence in drusen patients did not show any significant progression. However, we detected significant thinning of the surface nerve fibre layer over the optic nerve head in the central, superior and inferior segments. Due to the limitation of the axial resolution of the OCT it was not possible to perform a reliable assessment of the entire ONHD and to identify the lower border of the drusen. Since the total optic disc protrusion did not changed and RNFL thickness was reduced with the drusen are likely to become more visible as they become located more superficially with time which is in agreement with slowly progressive increase in the size and the number of visible drusen with increasing age seen on fundoscopy in previous studies [29, 30].

Thinning of the surface nerve fibre layer over the optic nerve head was more noticeable in the central, superior and inferior areas after 1 year. These areas correspond to the location of the inferior and superior vascular arcades. Previous literature has described changes in the vasculature of patients with ONHD including pronounced tortuosity, dilated veins [8], larger artery diameters with longer veins and vein branching close to the optic nerve [31] and possible alterations in the superficial retinal capillary network [32] as compared to the general population. In a recent publication of Cennamo et al. patients with ONHD were found to have a low flow index and reduced vessel density on OCT angiography [33]. It is possible, therefore that vascular changes contribute to the significant thinning of the surface nerve fibre layer we observed in the proximity of the major vessels arcades are the result of these previously described vascular changes. Patients showed significant progressive mean and nasal RNFL thinning over 12 months which is faster than previously believed based on fundoscopic assessment [30]. Although, changes we observed were statistically significant they were too small to be visible on inspection of the OCT scans or clinically. Previously Lee et al. indicated that ONHD are more often located on the nasal part of the optic disc [34]. Malmqvist et al. confirmed that RNFL thinning corresponds to the location of the ONHD in the optic nerve head [35]. It is possible therefore, that the more apparent nasal RNFL thinning we observed may be the result of the locations of the ONHD in the ONH which more commonly occur nasally. Interestingly, Malmqvist et al. reported that severe progression in size and number of drusen observed using superficial disc anatomy in 8 participants over a median of 56 years (mean age at initial and follow-up examination was, respectively, 16.8 and 73.3 years) [30]. This study was commenced prior to the advent of OCT technology. Here we demonstrate that using high resolution 3-dimensional imaging with OCT, changes in the papillary region can be documented over a much shorter period of time.

It would be interesting to assess optic nerve structure in this cohort of the patients over a longer follow-up period using OCT.

### Macular measurements

In previous studies patients with ONHD were shown to have RNFL and ILP thinning in the macular region compared to a control group [33, 36, 37]. This is the first study to investigate the within subject differences in the macular structure occurring over a 12 month period using OCT. Our study showed progressive thinning of the retina in the central, inner and outer temporal, outer superior and outer inferior areas of the macula. This was associated with a decrease of the RNFL thickness in the same areas excluding the outer superior quadrant of the macula. RNFL loss is described in different neuropathies [38–41] and is usually non-specific. Retinal layer subanalysis also showed significant reduction of the outer retina (ONL in the central retina and OS layer nasally) on the second visit. Similarly, to the optic disc changes, progression of the retinal parameters did not correlate with age and was not more pronounced in younger subject.

Potential limitations of this study are that the follow-up period was relatively short. Longer follow-up may help to better understand extend and severity of progression within patients with ONHD as well as providing clarity on the effect of patient age. In addition, the limited axial resolution of the OCT machine caused difficulties in the assessment of the part of the ONHD proximal to the lamina cribrosa.

For this study we selected participants with no field defects, in further studies it would be interesting to assess OCT changes in field loss group and how they correlate with different clinical and demographic characteristics.

New methods have recently been developed to determine the shape, location, and volume of buried ONHD using spectral-domain OCT [42]. Swept-source OCT also now provides the opportunity of enhanced depth imaging and more rapid acquisitions [43]. The development of the robust protocols with the use of newest OCT advancements could use these methods and technologies to track longitudinal change in ONHD with time.

In summary, our findings show that patients with ONHD demonstrate progressive thinning of the surface nerve fibre layer *(*hyperreflective space) and mean/nasal ppRNFL, thinning of the macula but unchanged disc protrusion. These changes could be detected using OCT in a short period of only 12 months which is faster that has been previously documented based on fundus photography data. Changes were found to be more pronounced in the proximity of the vascular arcades or disc drusen which may explain these changes, possibly through abnormal vessels or blood flow. Macular thinning found in ONHD has previously been associated with RNFL thinning in various other optic neuropathies. We did not find that the age of the patient was correlated to the magnitude of changes using OCT.

## Summary

### What was known before

- Several research groups reported in cross-sectional studies that ONHD increase in size with age on funduscopic examination.

### What this study adds

- Statistically detectable thinning of the optic nerve and macula structures occurred already after 12 months.

## Supplementary information

Supplementary table 1

Supplementary table 2

Supplementary figure 1

Supplementary figure 2

Supplementary subfigures

## Data Availability

Data is available on request.
