# Supplementary material for: Short-term progression of optic disc and macular changes in optic nerve head drusen
Source: Eye (Lond). 2022 Jul 16;37(7):1496–502. doi: 10.1038/s41433-022-02155-7 (PMC10169844; doi:10.1038/s41433-022-02155-7)
Supplement: Supplementary file 1 — Supplementary table 1 [file 41433_2022_2155_MOESM1_ESM.docx]

**Supplementary table 1:** Clinical and demographic characteristics of the patients with optic nerve head drusen, participated in the study.

| **ID** | **Age** | **Sex** | **Ethnicity** | **Affected eye** | **Vision (logMAR)** | | **Refraction (S.E.)** | | **IOP** | |
| --- | --- | --- | --- | --- | --- | --- | --- | --- | --- | --- |
|  |  |  |  |  | **RE** | **LE** | **RE** | **LE** | **RE** | **LE** |
| 1 | 46 | M | C | BE | -0.1 | -0.1 | -2.75 | -2.75 | 16 | 14 |
| 2 | 53 | F | C | BE | 0.0 | -0.1 | plano | plano | 13 | 15 |
| 3 | 43 | F | C | BE | 0.2 | 0.1 | plano | plano | 17 | 17 |
| 4 | 65 | F | C | BE | 0.3 | 0.1 | plano | plano | 18 | 20 |
| 5 | 74 | F | A | BE | 0.1 | 0.1 | +2.00 | +2.00 | 14 | 16 |
| 6 | 53 | M | A | BE | 0.1 | 0.1 | plano | plano | 20 | 20 |
| 7 | 21 | M | A | BE | 0.1 | 0.1 | +2.00 | +2.00 | 14 | 12 |
| 8 | 66 | F | C | BE | -0.1 | 0.0 | +1.00 | +1.00 | 12 | 12 |
| 9 | 37 | F | C | BE | 0 | 0.20 | plano | plano | 18 | 18 |
| 10 | 8 | F | C | BE | -0.1 | -0.1 | plano | plano | 16 | 17 |
| 11 | 70 | M | C | BE | 0 | 0 | plano | plano | 21 | 20 |
| 12 | 72 | M | C | BE | -0.1 | -0.1 | plano | plano | 19 | 19 |
| 13 | 55 | F | C | BE | -0.1 | 0 | +1.0 | plano | 14 | 13 |
| 14 | 36 | F | C | BE | -0.1 | -0.1 | plano | plano | 18 | 18 |
| 15 | 16 | F | C | BE | 0.2 | 0.1 | -1.25 | -1.25 | 19 | 18 |
| 16 | 55 | F | C | BE | 0.0 | 0.1 | -1.75 | -1.25 | 17 | 19 |
| 17 | 50 | M | C | BE | 0.2 | 0.1 | plano | plano | 11 | 12 |
| 18 | 21 | F | C | BE | -0.1 | -0.1 | plano | plano | 20 | 19 |
| 19 | 51 | M | C | BE | -0.1 | -0.1 | plano | plano | 18 | 18 |
| 20 | 62 | F | C | BE | 0.0 | 0.1 | plano | plano | 14 | 16 |
| 21 | 19 | F | C | BE | -0.1 | 0.0 | plano | plano | 17 | 17 |
| 22 | 17 | M | C | BE | -0.1 | 0.0 | plano | plano | 20 | 20 |
| 23 | 41 | F | C | BE | -0.1 | 0.0 | plano | plano | 16 | 18 |
| 24 | 13 | F | C | BE | -0.1 | 0.0 | plano | plano | 20 | 20 |
| 25 | 10 | F | C | BE | -0.1 | 0.0 | plano | plano | 14 | 14 |
| 26 | 54 | F | C | BE | 0.0 | 0.0 | plano | plano | 19 | 20 |
| 27 | 56 | F | C | BE | 0.0 | 0.1 | plano | plano | 20 | 20 |
| 28 | 66 | M | C | BE | 0.0 | 0.0 | plano | plano | 16 | 16 |
| 29 | 26 | M | C | BE | -0.1 | 0.0 | plano | plano | 16 | 17 |
| 30 | 31 | M | C | BE | 0.0 | 0.0 | plano | plano | 20 | 20 |
| 31 | 69 | M | C | BE | 0.1 | 0.1 | plano | plano | 17 | 17 |
| 32 | 32 | M | C | BE | -0.1 | 0.0 | plano | plano | 14 | 15 |
| 33 | 46 | M | C | BE | 0.0 | 0.0 | plano | plano | 17 | 16 |
| 34 | 46 | F | C | BE | 0.0 | 0.1 | plano | plano | 20 | 20 |
| 35 | 19 | M | C | BE | 0.0 | 0.1 | plano | plano | 19 | 18 |

M=male, F=female; RE=right eye, LE=left eye, BE=both eyes; S.E. = spherical equivalent.
