# Supplementary material for: Short-term progression of optic disc and macular changes in optic nerve head drusen
Source: Eye (Lond). 2022 Jul 16;37(7):1496–502. doi: 10.1038/s41433-022-02155-7 (PMC10169844; doi:10.1038/s41433-022-02155-7)
Supplement: Supplementary file 2 — Supplementary table 2 [file 41433_2022_2155_MOESM2_ESM.docx]

**Supplementary table 2:** Repeatability analysis of the optic nerve protrusion analysis in 13 participants on whom repeated optical coherence tomography scans have been carried out on the same visit.

|  |  |  |  |  |  |  |  |  |  |
| --- | --- | --- | --- | --- | --- | --- | --- | --- | --- |
|  | **Overall mean thickness (µm)** |  | **Mean difference (µm)** | **ULOA (µm)** | **LLOA (µm)** |  | **ICC** | **95% UCI** | **95% LCI** |
| Disc prominence |  |  |  |  |  |  |  |  |  |
| Centre (1mm diameter) | 514.3 |  | 0.885 | 3.718 | -1.949 |  | 1.000 | 1.000 | 1.000 |
| 1-3mm annulus |  |  |  |  |  |  |  |  |  |
| Temporal | 313.8 |  | 0.154 | 3.673 | -3.366 |  | 0.999 | 1.000 | 0.998 |
| Nasal | 349.4 |  | -0.577 | 2.767 | -3.921 |  | 1.000 | 1.000 | 0.999 |
| Superior | 384.5 |  | 1.154 | 4.894 | -2.586 |  | 1.000 | 1.000 | 0.999 |
| Inferior | 406.6 |  | -0.269 | 3.784 | -4.322 |  | 1.000 | 1.000 | 0.999 |
| Hyperreflective space | |  |  |  |  |  |  |  |  |
| Centre (1mm diameter) | 158.4 |  | 1.423 | 4.694 | -1.848 |  | 0.999 | 1.000 | 0.997 |
| 1-3mm annulus |  |  |  |  |  |  |  |  |  |
| Temporal | 116.5 |  | -0.615 | 1.982 | -3.213 |  | 0.999 | 1.000 | 0.997 |
| Nasal | 127.5 |  | 0.769 | 3.442 | -1.903 |  | 0.999 | 1.000 | 0.997 |
| Superior | 153.8 |  | 0.077 | 3.172 | -3.018 |  | 0.999 | 1.000 | 0.997 |
| Inferior | 170.0 |  | 0.962 | 5.652 | -3.729 |  | 0.998 | 0.999 | 0.992 |
|  |  |  |  |  |  |  |  |  |  |

ULOA = upper limit of agreement, LLOA = lower limit of agreement, ICC = intraclass correlation coefficient.
