# Supplementary figures and images for: Short-term progression of optic disc and macular changes in optic nerve head drusen
Source: Eye (Lond). 2022 Jul 16;37(7):1496–502. doi: 10.1038/s41433-022-02155-7 (PMC10169844; doi:10.1038/s41433-022-02155-7)

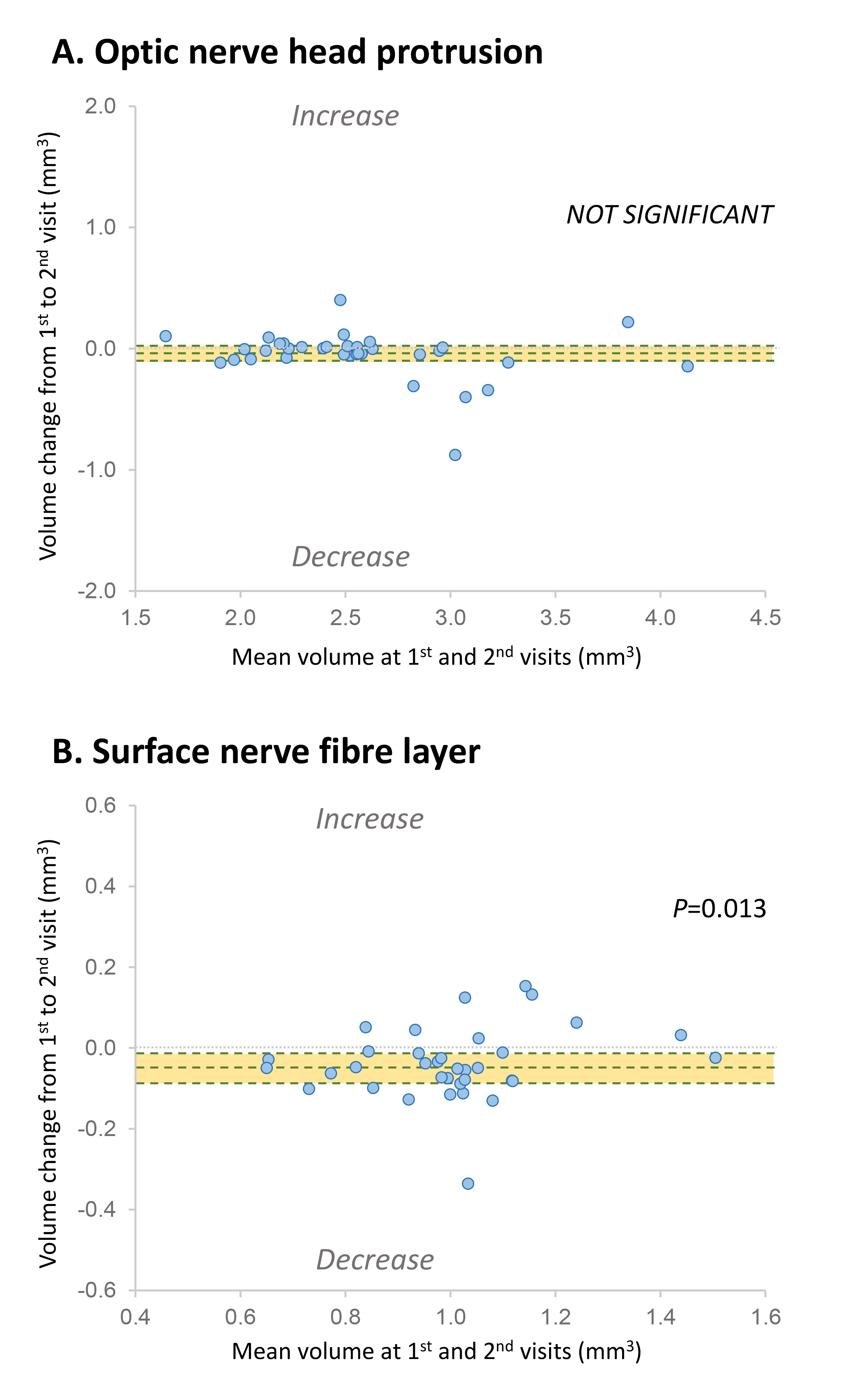

Supplement: Supplementary file 3 — Supplementary figure 1 [file 41433_2022_2155_MOESM3_ESM.tif]

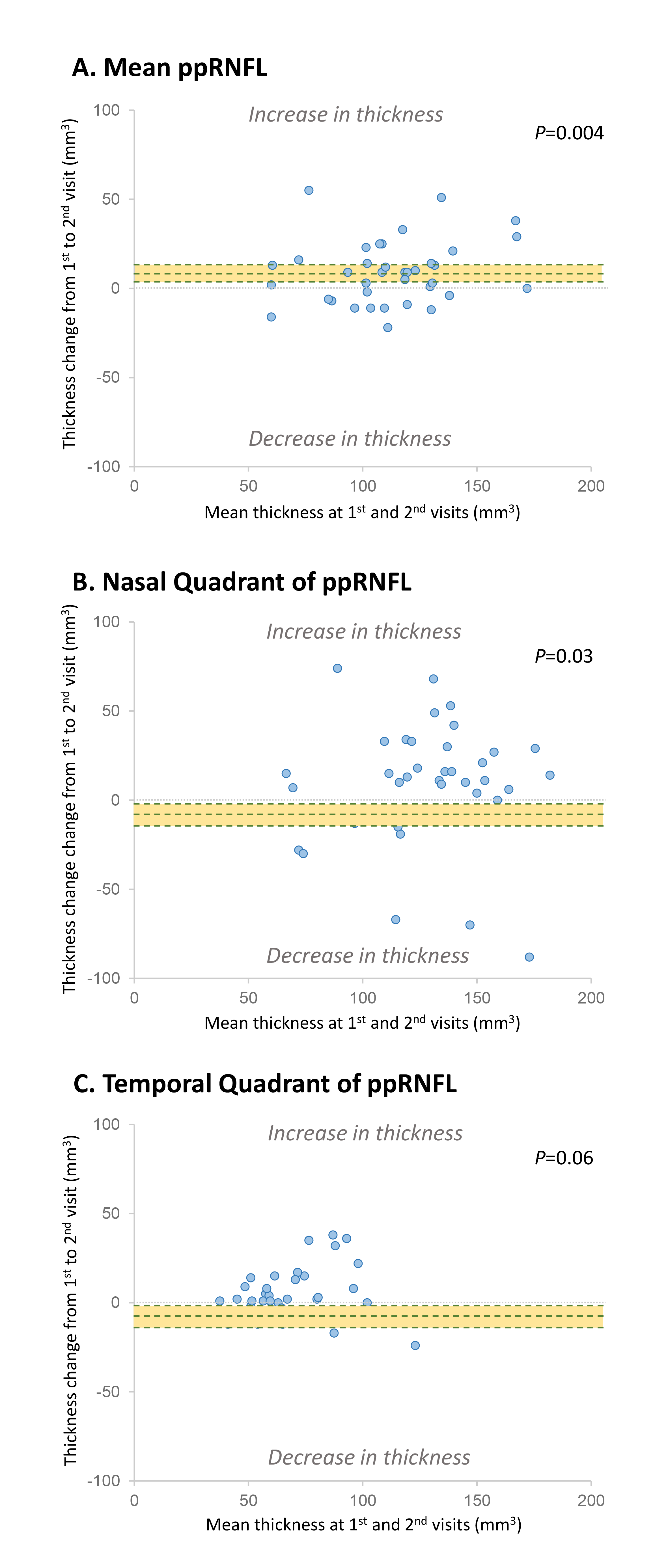

Supplement: Supplementary file 4 — Supplementary figure 2 [file 41433_2022_2155_MOESM4_ESM.tif]
