# Supplementary material for: Short-term progression of optic disc and macular changes in optic nerve head drusen
Source: Eye (Lond). 2022 Jul 16;37(7):1496–502. doi: 10.1038/s41433-022-02155-7 (PMC10169844; doi:10.1038/s41433-022-02155-7)
Supplement: Supplementary file 5 — Supplementary subfigures [file 41433_2022_2155_MOESM5_ESM.docx]

***Supplementary figure 1****: Bland-Altman plots of individual changes (blue circles) in area between first and second visits with respect to the mean area for the two visits for: (A) optic nerve head protrusion and (B) volume of the surface nerve fibre layer (µm) in patients with optic nerve head drusen. The mean change and 95% confidence interval of the mean is shown in yellow. Patients have significant thinning in surface nerve fibre layer (p=0.013).*

***Supplementary figure 2****: Bland-Altman plots of individual changes (blue circles) in ppRNFL thicknesses in mean (A), nasal (B) and temporal (C) quadrants. The mean change and 95% confidence interval of the mean is shown in yellow. Patients have significant thinning in mean (p=0.004) and nasal (p=0.028) ppRNFL quadrant.*
